# Three strains isolated from Northern Germany constitute the novel genera *Njordella* and *Rania* in the family *Pirellulaceae*

**DOI:** 10.1038/s41598-026-55534-5

**Published:** 2026-06-04

**Authors:** Myriel Staack, Tom Haufschild, Jonathan Hammer, Nicolai Kallscheuer, Gaurav Kumar, Christian Jogler

**Affiliations:** 1 Department of Microbial Interactions, Institute of Microbiology, Friedrich-Schiller-University, Jena, Germany; 2 Cluster of Excellence Balance of Microverse, Friedrich-Schiller-University, Jena, Germany

**Keywords:** *Pirellulaceae*, Schlesner strain collection, Marine bacteria, Asymmetric cell division, Baltic Sea, Ecology, Ecology, Genetics, Microbiology

## Abstract

This study reports the characterization of three novel species belonging to the two newly established genera *Njordella* gen. nov. and *Rania* gen. nov. in the family *Pirellulaceae* within the phylum *Planctomycetota*. The three investigated strains SH302^T^, SH451^T^ and SH573^T^ were isolated from water samples originating from distinct locations across Northern Germany: Lägerdorf (a municipality close to Hamburg), Fjord Schlei (a 42 km estuary of the Baltic Sea) and Fehmarn Island in the Baltic Sea. All strains form (light) pink colonies, reproduce asymmetrically by polar budding and display an acorn-shaped cell morphology typical in the family *Pirellulaceae*. Rosette formation was only observed for strains SH302^T^ and SH573^T^. The three strains are mesophilic with a temperature optimum at 28 °C, aerobic and heterotrophic, but differ in their pH preference. While strains SH302^T^ and SH573^T^ are neutrophilic (pH optimum at 7.0 and 7.5, respectively), strain SH451^T^ is slightly alkaliphilic (optimal growth at pH 8.0). The genome sizes of strains SH302^T^, SH451^T^ and SH573^T^ are 7.2 Mb, 6.6 Mb and 9.4 Mb, respectively. Based on phylogenetic, morphological and physiological analyses, we conclude that the novel strains belong to three novel species of two novel genera. For the novel taxa, we introduce the names *Njordella aestuarii* gen. nov., sp. nov., represented by strain SH451^T^ (= CECT 30907^T^ = KCTC 102089^T^) as the type strain and *Njordella calcicola* sp. nov. with type strain SH302^T^ (= DSM 116728^T^ = KCTC 102091^T^). For the third taxon, we chose the name *Rania fehmarnensis* gen. nov., sp. nov. represented by the type strain SH573^T^ (= DSM 116763^T^ = CECT 31080^T^ = KCTC 102135^T^).

**Supplementary Information:**

The online version contains supplementary material available at 10.1038/s41598-026-55534-5.

## Introduction

With an ubiquitous distribution across natural habitats, strains belonging to the phylum *Planctomycetota* play key roles in carbon and nitrogen cycling^1^. While the phylum ranks fourth in the list of the most abundant bacterial phyla in soil, our research largely focuses on aquatic environments, in which strains belonging the phylum are often found associated to phototrophs^2–7^. They can even dominate biotic surface-associated biofilms^8,9^ in which they degrade high molecular weight carbon substrates such as recalcitrant polysaccharides^10–12^. The uptake of polysaccharide molecules is currently under study and may involve dedicated fibers that originate from crateriform structures; the latter being a unique feature of planctomycetal outer membranes^13^. Invaginations of the inner membrane lead to a highly enlarged periplasm that potentially acts as a bacterial “stomach” during the breakdown of intact internalized polysaccharide molecules^13^. If true, planctomycetes follow a more “selfish” strategy for getting sources of carbon and energy compared to the more common secretion of exoenzymes for macromolecule degradation outside of the cell.

In general, the phylum *Planctomycetota* questions classical concepts of bacterial biology. Notable examples include the shape-shifting *Saltatorellus* clade^14^ and the genus “*Candidatus* Uabimicrobium”^15,16^. Strains belonging to the latter are obligate predatory bacteria (“bacteria of prey”) which internalize entire bacterial cells as prey via an uncharacterized phagocytosis-like uptake mechanism. Such findings reflect the unusual cell biology of the phylum, which sets planctomycetes apart from canonical Gram-negative bacteria^13,17^. Unlike most bacteria, planctomycetes divide by asymmetric cell division (“budding”) instead of binary fission^18^. Some strains follow a dimorphic lifestyle: a sessile stalked mother cell divides and the daughter cell, a planktonic swimmer, can colonize new surfaces^19^. Interestingly, planctomycetes lack most of the canonical divisome proteins, including the otherwise universal cell division protein FtsZ^18,20^. Only the gene encoding the DNA translocase FtsK appears to be essential in this phylum, suggesting a fundamentally altered mechanism of bacterial cell division^21,22^. Although peptidoglycan presence has been validated in planctomycetal cell walls^17,23^, proteins known to be involved in connecting the peptidoglycan monomers into the sacculus are not functioning as described in other model bacteria^20,24,25^. Furthermore, canonical peptidoglycan synthesis genes unexpectedly appear to be non-essential^22^.

To date, all sequenced genomes of strains belonging to the phylum harbor biosynthetic gene clusters (BGCs)^20,26,27^ with a high percentage of predicted regions having no similarity with known clusters in the MIBiG (Minimum Information about a Biosynthetic Gene cluster) database, highlighting their potential as yet for the most part untapped producers of bioactive small molecules^26,28–31^. Characterized examples include stieleriacines (potential biosurfactants)^32–34^, carotenoid pigments^35,36^, an aromatic plant toxin^37^ and alkylresorcinols of yet unknown function^38^. The actual biosynthetic potential is likely even higher than predicted by common bioinformatic tools since functional gene identification in planctomycetal genomes is challenging, because this phylum contains more genes of unknown function than any other group of bacteria^39^.

Metagenome and amplicon sequencing studies consistently demonstrate the high abundance and broad distribution of *Planctomycetota* across a wide range of ecosystems including permafrost, sea and glacial ice, oxygen minimal zones and marine deep surfaces, yet many lineages remain uncultured, making the recovery and cultivation of representative strains a central challenge^40–48^. We and other groups isolated and characterized additional *Planctomycetota* representatives from distinct habitats. This includes sampling sites ranging from Monterey Bay (USA) kelp forests^49^, the macroalga *Fucus spiralis*^50,51^, algae in hydrothermal environments^52–54^, Mediterranean Sea *Posidonia* seagrass meadows^9^, hydrothermal vents^52,55–57^, marine active volcanic sites^58–60^, plastic particles^61,62^, wood specimens^63^, marine microbial mats^59,64^, limnic cyanobacterial blooms^65^, jellyfish^66^, marine^67–69^ and limnic sponges^70^, sediments of seawater fishtanks^71^, wastewater^72^, subsurface water^73^ to termite guts^74^.

Here, we report the characterization of three novel strains, SH302^T^, SH451^T^ and SH573^T^, originating from the strain collection of Heinz Schlesner^75^ from the 1980s, which define two new genera within the family *Pirellulaceae*.

## Material and methods

### Sampling and strain isolation

All three strains, SH302^T^, SH451^T^ and SH573^T^, were isolated from water samples of distinct sources in Northern Germany. Strain SH302^T^ was sampled in a chalk mine in Lägerdorf in Schleswig–Holstein, Germany (53.882119° N, 9.568021° E) prior to 1999^76,77^. Strain SH451^T^ was sampled from Fjord Schlei, an estuary of the Baltic Sea^77^, Germany (54.595374° N, 9.844947° E) prior to 2004. Strain SH573^T^ was sampled at the creek Kopendorfer Au at Fehmarn Island^77^, Germany (54.470831° N, 11.092419° E) prior to 2004. Comprehensive descriptions of the isolation methods are provided in studies supervised by Heinz Schlesner^76,78^.

All isolated strains were initially cryopreserved by Heinz Schlesner and later revived from the stocks. Cultivation of the strains was performed using two distinct media: M30aPY (pH 8.0) for strains SH302^T^ and SH451^T^ and M1aPY (pH 7.0) for strain SH573^T^. Preparation of the media followed previously established protocols^77^. Unless otherwise stated, all cultures were incubated at 21 °C. Cryopreservation of the axenic cultures was performed using M30aPY or M1aPY medium supplemented with 50% (vol/vol) glycerol and cryogenic stocks were stored at − 80 °C.

### Light microscopy

Light microscopy and image analysis were conducted as described earlier^72^. In summary, cultures at the half-maximal optical density at 600 nm (OD_600_) (SH302^T^ = 0.5, SH451^T^ = 1.0, SH573^T^ = 1.2) were spotted onto a 1% (w/v) agarose cushion that subsequently solidified. The mount was covered with a coverslip and movement was prevented by the application of VLAP (33% vaseline, 33% lanolin, 33% paraffin, w/w). Cells were imaged in an inverse Nikon Ti2 microscope using a Nikon Plan Apo λ 100 × immersion oil objective with a phase ring for phase contrast (PhC) images and without a phase ring for differential interference contrast (DIC) imaging. Images were captured with a Nikon DS-Ri2 camera and the NIS-Elements software (version 5.30). Native files were transformed into single-channel TIFF RGB images in FIJI^79^, which were analyzed using BacStalk^80^ with 25 and 15 pixels for cell size and minimum cell size, respectively for segmentation settings. Three replicates were analyzed summing up to 450 cells per strain in total. The obtained data was visualized with SuperPlotsOfData^81^. Brightness and contrast were adjusted manually in FIJI for visualization purposes only.

### Determination of temperature and pH optimum for growth

The optimal growth temperature was assessed based on the colony-covered surface area on agar plates incubated at nine different temperatures (4, 10, 18, 21, 24, 28, 32, 37, 42 °C) in biological duplicates. Growth performance was visually inspected, photographically documented and qualitatively classified as follows: no growth (-), moderate growth (+), good growth (++), very good growth (+++). The determination of growth at the optimal pH was performed in M30aPY/M1aPY medium, buffered to pH values of 5.0, 6.0, 7.0, 7.5, 8.0, 9.0, and 10.0 using 100 mM of the appropriate buffer (2-(*N*-morpholino) ethanesulfonic acid (MES), 3-[4-(2-hydroxyethyl)piperazin-1-yl]propane-1-sulfonic acid (HEPES), or *N*-cyclohexyl-2-aminoethanesulfonic acid (CHES)), according to their recommended buffering ranges. The OD_600_ of the cultures was measured at the previously determined optimal temperature using the microplate reader Epoch2 (BioTek) in biological duplicates and technical triplicates. The lid was maintained at 2 °C above the incubation temperature to reduce evaporation.

### Genome sequencing and assembly

Genomic DNA was isolated and used for Oxford Nanopore as well as Illumina sequencing as described^72^ with modifications previously reported^82^. Basecalled reads were uploaded to the Galaxy web-based platform and the server available under the public domain usegalaxy.eu^83^ to execute the bioinformatic workflows for genome assembly and polishing^72^. The sequencing chemistry and bioinformatic tools including tool version numbers and optional parameters are listed in Table S1. The Prokka-annotated chromosome was re-oriented to the start codon of the *dnaA* gene encoding the replication initiator protein^84^, and finally re-annotated using PGAP version 2025–05-06, build 7983^85^. CheckM version 1.2.3^86^ was used to assess genome completeness and to evaluate coding density and DNA G + C content.

### Nucleotide accession numbers

The 16S rRNA gene sequences of the novel strains are available from GenBank under the accession numbers PX530357 (SH302^T^), PX530377 (SH451^T^) and PX530413 (SH573^T^). Genome sequences have been deposited at DDBJ/ENA/GenBank under the accession numbers JBWOZO000000000 (SH302^T^), JBWOZP000000000 (SH451^T^) and JBWOZQ000000000 (SH573^T^).

### Phylogenetic analyses

The 16S rRNA gene sequences of strains SH302^T^, SH451^T^, SH573^T^ and all described representatives of the phylum *Planctomycetota* as listed in the List of Prokaryotic names with Standing in Nomenclature (LPSN, accessed on January 12th, 2026) were retrieved from the annotated genomes and aligned using ClustalW^87^. Based on this alignment, a 16S rRNA gene sequence similarity matrix was generated using TaxonDC^88^. A maximum likelihood phylogenetic tree was constructed in FastTree 2.1^89^ based on the same alignment by applying the GTR + CAT model with 1000 bootstrap replications. The following three 16S rRNA gene sequences from bacterial type strains clustering outside of the *Planctomycetota*, but part of the *Planctomycetota-Verrucomicrobiota-Chlamydiota* (PVC) superphylum, were used as outgroup: *Opitutus terrae* (NCBI accession number AJ229235), *Kiritimatiella glycovorans* (acc. no. NR_146840) and *Lentisphaera araneosa* (acc. no. NR_027571). The multi-locus sequence analysis (MLSA)-based phylogenetic analysis was computed via autoMLST using 500 bootstrap replicates^90^. Using the NCBI reference genomes of all recognized type strains of the family *Pirellulaceae,* this analysis was carried out with the autoMLST-simplified-wrapper tool (available on GitHub). The genomes of three strains of the family *Planctomycetaceae* served as outgroup: *Planctopirus limnophila* DSM 3776^T^ (acc. no. GCF_000092105.1), *Rubinisphaera brasiliensis* DSM 5305^T^ (acc. no. GCA_000165715.3) and *Planctomicrobium piriforme* P3^T^ (acc. no. GCA_900113665.1). The determination of average amino acid identities (AAI) and average nucleotide identities (ANI) was performed using the respective scripts of the enveomics collection^91^, while the percentage of conserved proteins (POCP) was computed according to the published protocol^92^. Sequences of the *rpoB* gene encoding the *β*-subunit of the DNA-directed RNA polymerase were extracted from publicly available online databases and sequence identities of the suggested 1298 bp partial sequence were determined following previously established protocols^93,94^.

### Analysis of genome-encoded features

Putative carbohydrate-active enzyme (CAZyme)-encoding genes were analyzed with dbCAN3^95^. The in silico prediction of BGCs putatively involved in the biosynthesis of secondary metabolites was conducted using the web version of antiSMASH 7 with relaxed strictness and all extra features activated^96^.

## Results and discussion

### Phylogenetic analyses

**Fig. 1:**
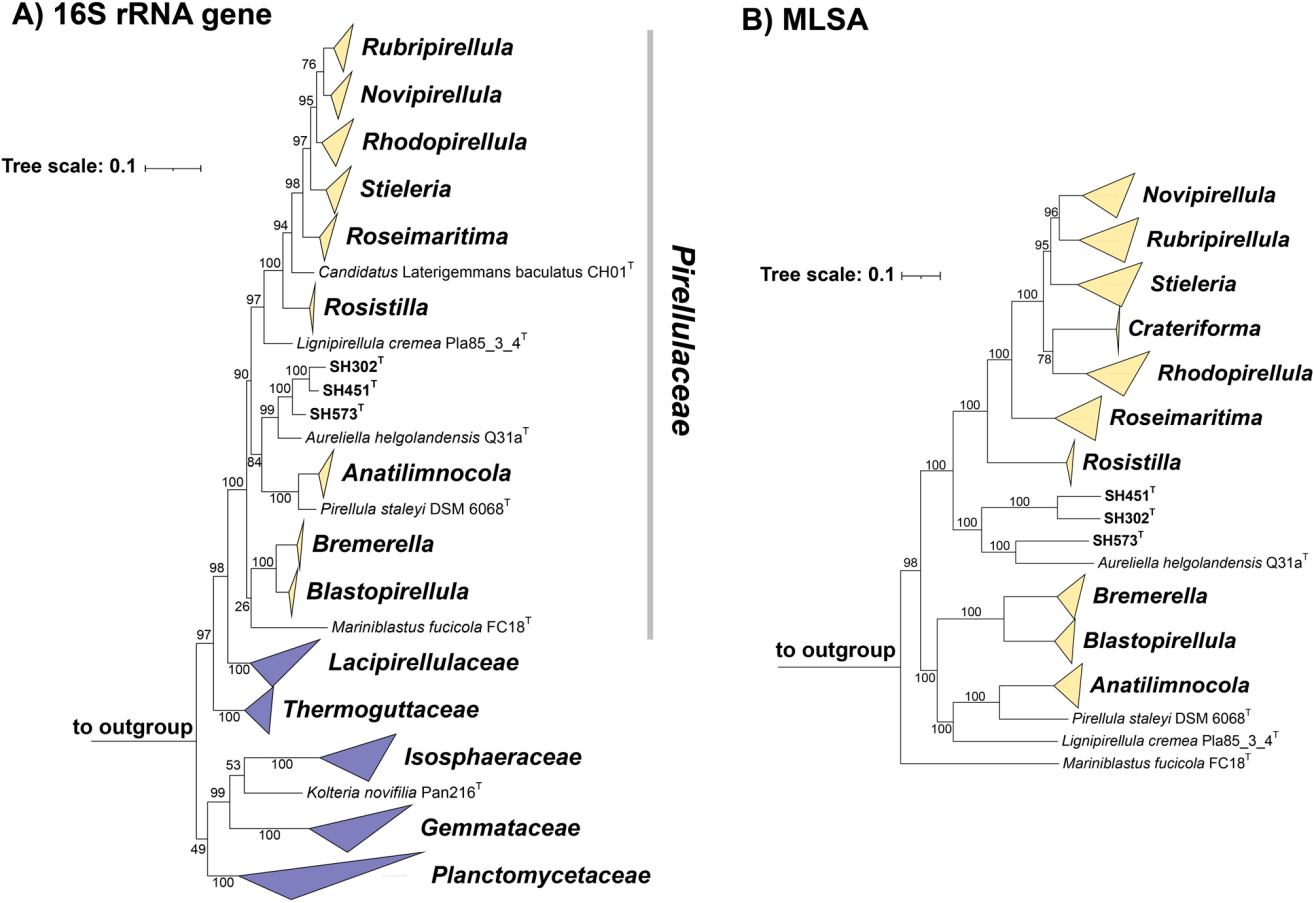
16S rRNA gene sequence- and MLSA-based phylogenetic trees. The maximum likelihood phylogenetic trees illustrate positions of the strains SH302^T^, SH451^T^ and SH573^T^ in the family *Pirellulaceae*. Bootstrap values based on 1000 re-samplings for 16S rRNA gene sequences (**A**) and 500 re-samplings for MLSA (**B**) are indicated at the nodes (in %). Outgroup sequences are provided in the Material and methods section. The scale bar represents the number of substitutions per nucleotide (16S rRNA gene sequence-based tree) or amino acid position (MLSA-based tree).

Phylogenetic analyses based on 16S rRNA gene sequences and MLSA position the three strains SH302^T^, SH451^T^ and SH573^T^ in the family *Pirellulaceae* (Fig. 1). In the 16S rRNA gene sequence-based tree, the strains SH302^T^ and SH451^T^ formed a distinct clade, while SH573^T^ branched separately. MLSA analyses confirmed the closer relationship of strains SH302^T^ and SH451^T^, whereas strain SH573^T^ clustered with *Aureliella helgolandensis* Q31a^T^ on a neighboring lineage. Based on the clustering pattern in both trees, the three strains were compared with *A. helgolandensis* Q31a^T^ using an established set of single gene- and whole genome-based phylogenetic markers. This extended comparison was necessary because reliance on 16S rRNA gene sequence similarity alone has previously proven insufficient for robust species delineation within the phylum *Planctomycetota*^70^.

**Fig. 2:**
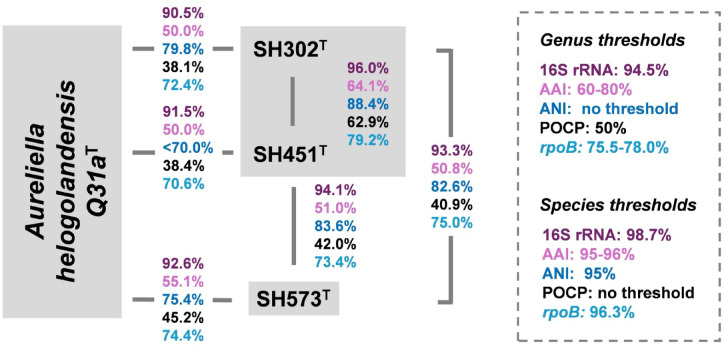
Comparison of phylogenetic markers. The strains SH302^T^, SH451^T^, SH573^T^ and *Aureliella helgolandensis* Q31a^T^ were analyzed using several established phylogenetic markers, including 16S rRNA gene sequence similarity (16S rRNA), average amino acid identity (AAI), average nucleotide identity (ANI), percentage of conserved proteins (POCP) and partial *rpoB* gene sequence similarity (*rpoB*).

In the all-versus-all comparison, the maximal 16S rRNA gene sequence similarity of 96.0% between strains SH302^T^ and SH451^T^ fell above the genus-level cutoff of 94.5%^97^, whereas all other combinations yielded values below the genus threshold (Fig. 2). Whole-genome relatedness indices reinforced these findings: strains SH302^T^ and SH451^T^ yielded values for ANI of 88.4% and for AAI of 64.1%, consistent with their affiliation to the same genus but distinct species^98^. The POCP value of 62.9% also exceeded the genus cutoff, further supporting the classification^92^. By contrast, SH573^T^ exhibited a 16S rRNA gene sequence similarity below 93.3%, AAI values below 60–80% and POCP values below 50% when compared to strains SH302^T^, SH451^T^ and *A. helgolandensis* Q31a^T^, indicating placement in a distinct genus. The single-gene marker *rpoB* reinforced this finding, suggesting that only the two strains SH302^T^ and SH451^T^ belong to the same genus based on sequence identities (79.2%, genus threshold of 75.5–78.0%), while the other analyzed strains fall clearly below the threshold^93^.

For strain SH573^T^ all comparisons of 16S rRNA gene sequences gave similarity values below the genus threshold of 94.5%. A maximal ANI value of 83.6% for comparison of any of the included strains with strain SH573^T^ excludes that it belongs to any previously described species. Whole-genome comparison even support the delineation from described genera; with AAI and POCP values below the genus threshold values (Fig. 2). These observations clearly identify strain SH573^T^ as representing not only a novel species, but also belonging to a novel genus within the family *Pirellulaceae*.

Taken together, the results indicate that strains SH302^T^ and SH451^T^ belong to two novel species of the same novel genus, whereas strain SH573^T^ belongs to a separate species of a separate genus.

### Analysis of genomic and genome-encoded features

Genome sequencing of strains SH451^T^ and SH573^T^ yielded closed genomes of 6.6 Mbp and 9.4 Mbp, respectively, with a completeness of 99.2% each. For strain SH302^T^ a closed genome of 7.2 Mb with a slightly lower completeness of 98.8% was obtained along with three linear contigs below 10 kbp. For comparison, the closest relative *A. helgolandensis* Q31a^T^ has a genome size of 8.4 Mb, placing it between the analyzed strains: its genome is smaller than that of strain SH573^T^, but larger compared to the genomes of strains SH302^T^ and SH451^T^. The proportion of hypothetical proteins encoded in the compared genomes falls in the range of 25–30% (based on the PGAP annotation). With 25.0%, *A. helgolandensis* Q31a^T^ is at the lower boundary of this range. Numbers of tRNA genes and coding density values in the four compared strains are similar. However, the numbers of (protein-coding) genes per Mbp differ significantly (Table 1). Especially strain SH573^T^, the strain with the largest genome, stands out with a lower number of (protein-coding) genes per Mbp compared to the other three strains. Since the coding density of this strain is even higher than in two of the four compared strains, this suggests that the average protein length in this strain should be higher. However, this was not further investigated at this stage. While *A. helgolandensis* Q31a^T^ harbors two copies each of 5S, 16S and 23S rRNA genes, these genes are present in single copy in the novel strains.

**Table 1:** Genomic and genome-encoded features of strains SH302^T^, SH451^T^ and SH573^T^ compared to *Aureliella helgolandensis* Q31a^T^.

Characteristics	SH302^T^	SH451^T^	SH573^T^	*Aureliella helgolandensis* Q31a^T^
Genomic features				
RefSeq accession number	GCF_056494125.1	GCF_056494165.1	GCF_056494185.1	GCF_007752135.1
Genome size (bp)	7,174,608	6,566,063	9,357,046	8,439,957
Contigs	4	1	1	1
Plasmids	Unclear	No	No	No
DNA G + C (%)	52.1	50.9	54.6	55.3
Completeness (%)	98.8	99.2	99.2	98.1
Genes	5556	5122	6325	6045
Genes/Mbp	774	780	676	716
Protein-coding genes	5465	5030	6217	5927
Protein-coding genes/Mbp	761	766	664	702
Hypothetical proteins*	1634	1424	1571	1484
Hypothetical proteins (%)	29.9	28.3	25.3	25.0
Coding density (%)	88.0	87.2	87.8	85.0
rRNA genes (5S,16S,23S)	1,1,1	1,1,1	1,1,1	2,2,2
tRNA genes	65	68	71	72
Secondary metabolite-associated biosynthetic gene clusters				
Terpene (incl. precursors)	7	4	3	2
Type I PKS	2	2	2	0
Mixed type I PKS-NRPS	2	1	0	0
Type II PKS-like	0	0	1	0
Type III PKS	0	1	0	1
Mixed type III PKS-NRPS	0	0	1	0
Ribosomally-synthesized peptide-related	0	0	1	0
NRPS-like	2	2	2	0
*N*-acetylglutaminylglutamine amide	0	1	0	0
Bacteriocin	0	0	0	1
Resorcinol	0	0	0	1
Others	0	0	0	2
BGCs (total)	13	11	10	7
BGCs per Mbp	1.8	1.7	1.0	0.8
Carbohydrate-active enzymes				
Glycoside hydrolases	44	61	60	52
Glycosyltransferases	77	57	65	73
Polysaccharide lyases	1	2	1	6
Carbohydrate esterases	28	24	22	12
Carbohydrate-bind. modules	23	26	28	16
Auxiliary activities	1	2	3	n/a
CAZyme genes (total)	174	172	179	159
CAZyme genes per Mbp	24	26	19	19

*based on the PGAP/Refseq-annotated genomes, PKS: polyketide synthase, NRPS: non-ribosomal peptide synthetase.

Strains in the class *Planctomycetia*, to which also the novel strains belong, have genome sizes in the range of 4.3–12.4 Mbp and typically harbor one BGC per Mbp. Since most of these BGCs are uncharacterized, the class potentially produces novel secondary metabolites with important bioactivities^30,99^. To explore this capacity, we mined the genomes for BGCs and compared the protein or compound class they are most likely encoding or associated with (Table 1). Strain SH302^T^ harbors the highest number of 13 BGCs, whereas strain SH451^T^ has eleven, SH573^T^ ten and the closest relative *A. helgolandensis* Q31a^T^ only seven BGCs. All three novel strains share a set of two type I PKSs and two NRPS-like proteins. Beyond this conserved set, the strains diverge considerably (Table 1). Strain SH302^T^ additionally harbors seven BGCs possibly involved in terpene production and two mixed type I PKS-NRPS gene clusters. Strain SH451^T^ harbors four terpene BGCs, one mixed type I PKS-NRPS, two NRPS-like and one *N*-acetylglutaminylglutamine amide BGCs. Strain SH573^T^ harbors only three terpene BGCs, as well as one type II PKS-like, one mixed type III PKS-NRPS and one ribosomally-synthesized peptide-related (RiPP) BGC. In contrast, *A. helgolandensis* Q31a^T^ harbors only seven BGCs putatively involved in the biosynthesis of terpenes, type III PKS-derived polyketides, bacteriocins, resorcinols and two additional unclassified clusters. Thus, the novel strains not only possess a greater number of BGCs than their closest relative, but BGCs also point towards differences in secondary metabolite repertoires, suggesting unexplored biosynthetic potential within the lineage. With numbers of BGCs per Mbp close to 2, strains SH302^T^ and SH451^T^ are enriched in BGCs since typically one BGC per Mbp is predicted in planctomycetal genomes.

To be successful in challenging environments, bacteria must develop adaptive traits that provide advantages over competitors. Studies have exposed the phylum *Planctomcyetota* to possess strong hydrolytic capacities, including enzymes involved in the degradation of (decorated) polysaccharides^100^. Genomic analysis of the three novel strains revealed a comparable repertoire of carbohydrate-active enzymes (CAZymes) with counts ranging from 172 in strain SH451^T^ over 174 in strain SH302^T^ to 179 in strain SH573^T^ (Table 1). These numbers are higher compared to the closest relative *A. helgolandensis* Q31a^T^, which harbors only 159 putative CAZyme-encoding genes. All strains encode similar numbers of polysaccharide lyases, carbohydrate esterases, carbohydrate-binding modules and enzymes involved in auxiliary activity. However, strain SH302^T^ harbors the highest number of glycosyltransferases but at the same time exhibits the lowest number of glycoside hydrolases. This CAZyme repertoire suggests a metabolic versatility, potentially providing these strains with an advantage in oligotrophic environments.

**Fig. 3:**
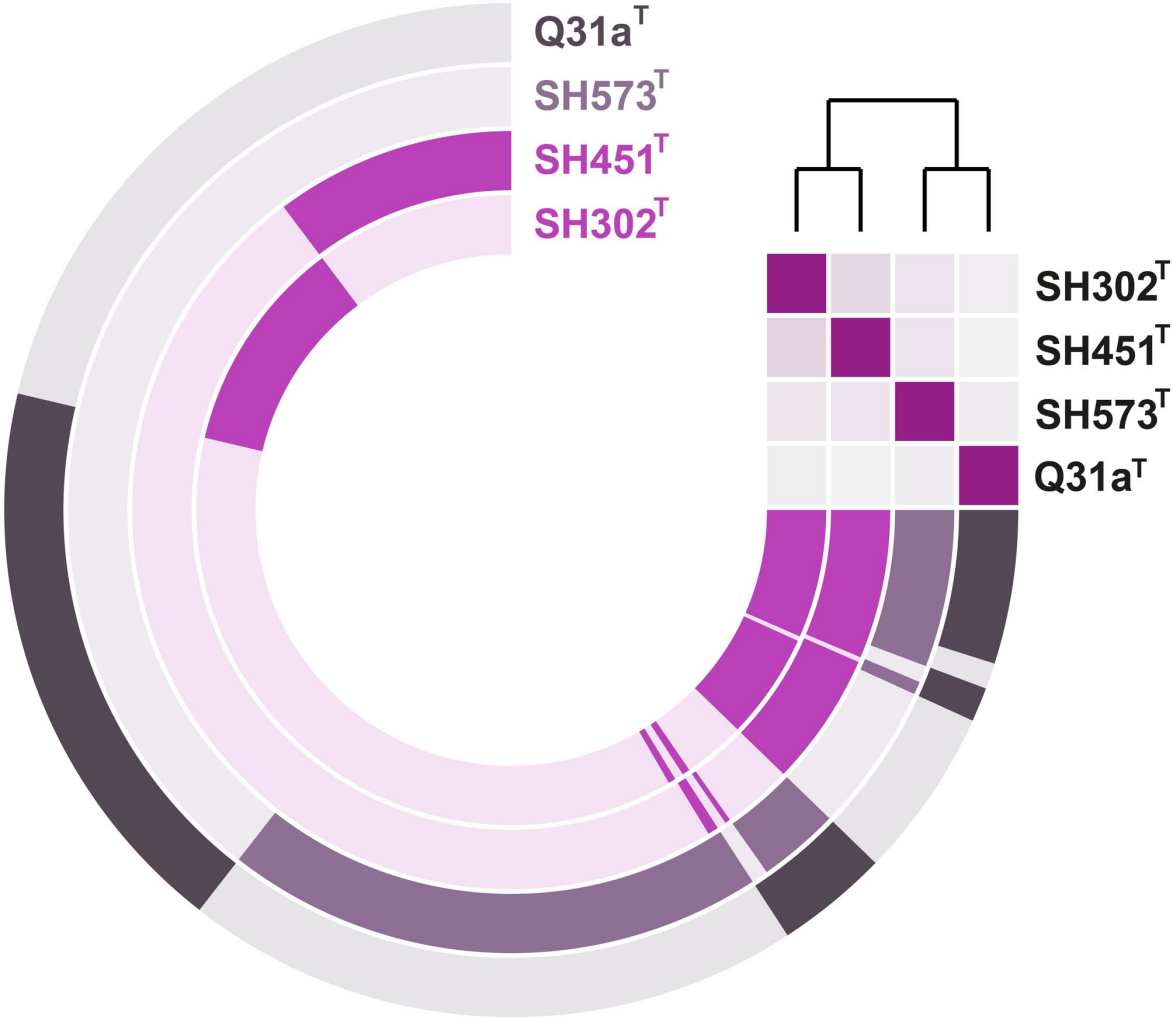
Pangenome of strains SH302^T^, SH451^T^, SH573^T^ and its closest relative *Aureliella helgolandensis* Q31a^T^. The concentric rings depict the gene content of the genomes analyzed. Dark segments correspond to shared genes, whereas light segments indicate genes without homologs in the other genome. Shades of purple were used to depict the three described strains, with two tones differentiating the distinct genera. The heatmap in the upper right corner shows genome relatedness among the strains based on ANI values (faint purple: ≤ 70% to bright purple: 100%).

The constructed pangenome of strains SH302^T^, SH451^T^, SH573^T^ and *A. helgolandensis* Q31a^T^ comprised 16,332 gene clusters across the four genomes (Fig. 3). Among these, only 1429 gene clusters were shared by all strains, representing the core genome and indicating a relatively small proportion of conserved genes. The number of strain-specific singletons was notably high, particularly in strain SH573^T^ with 4287 singletons, compared to 2384 in strain SH302^T^, 2218 in SH451^T^ and 3942 singletons in *A. helgolandensis* Q31a^T^. The high number of singletons is likely attributed to the limited number of genomes included in the pangenome analysis, however singletons can provide important insights into the strain-specific regulatory and metabolic repertoires, potential phage-derived sequences, specialized traits, etc.

### Characterization of basal morphological and physiological characteristics

**Fig. 4:**
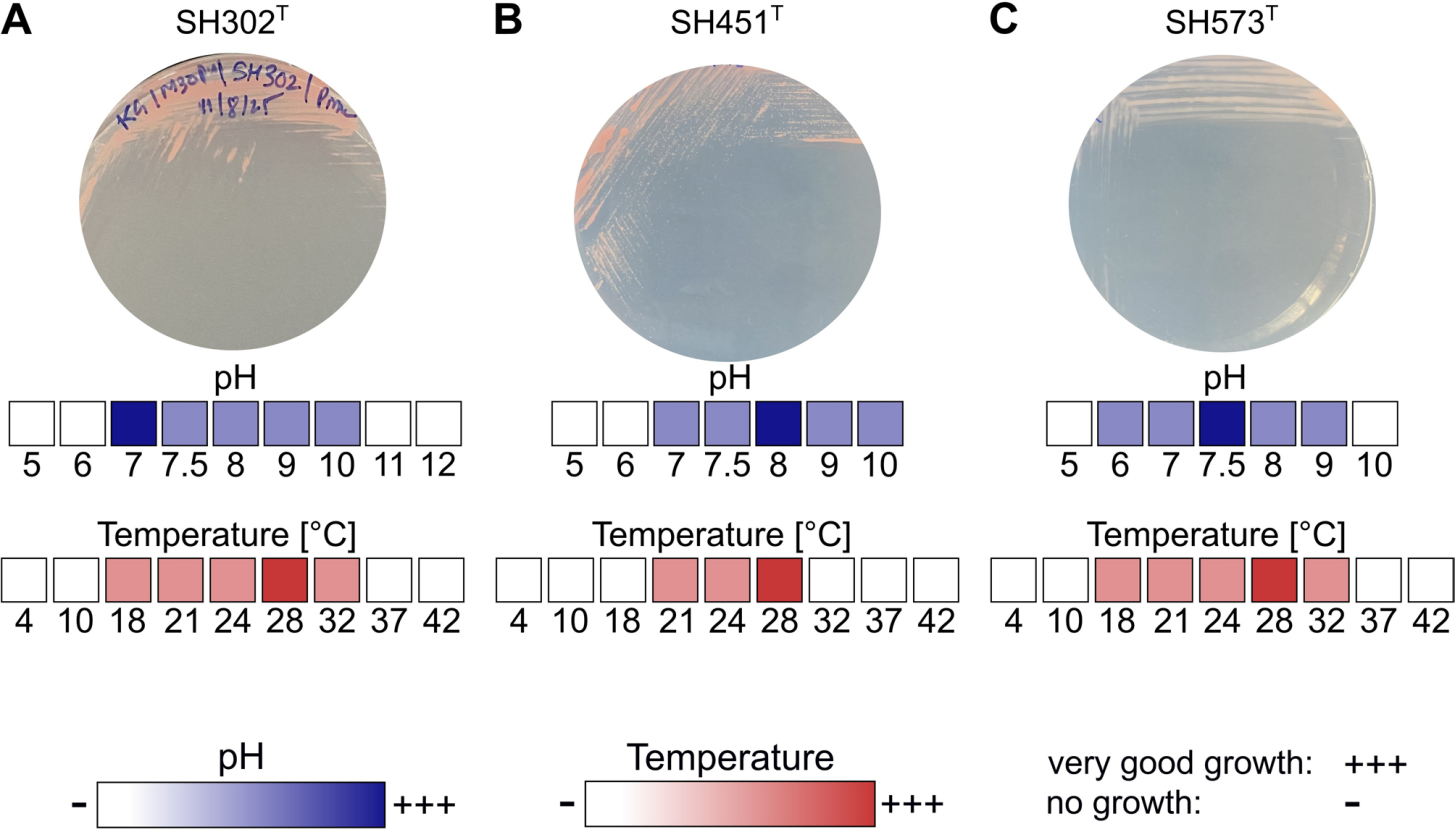
Phenotypic characterization of strains SH302^T^, SH451^T^ and SH573^T^. Colony morphology of each strain on M30aPY (SH302^T^ and SH451^T^) and M1aPY (SH573^T^) agar plates (**A**–**C**). Qualitative growth assessment of strains at varying pH values (5.0–10.0 or 5.0–12.0) and temperatures (4–42 °C). The growth intensity is represented by a color gradient in blue (pH) and red (temperature), ranging from light (white, no growth) to dark (very good growth).

**Fig. 5:**
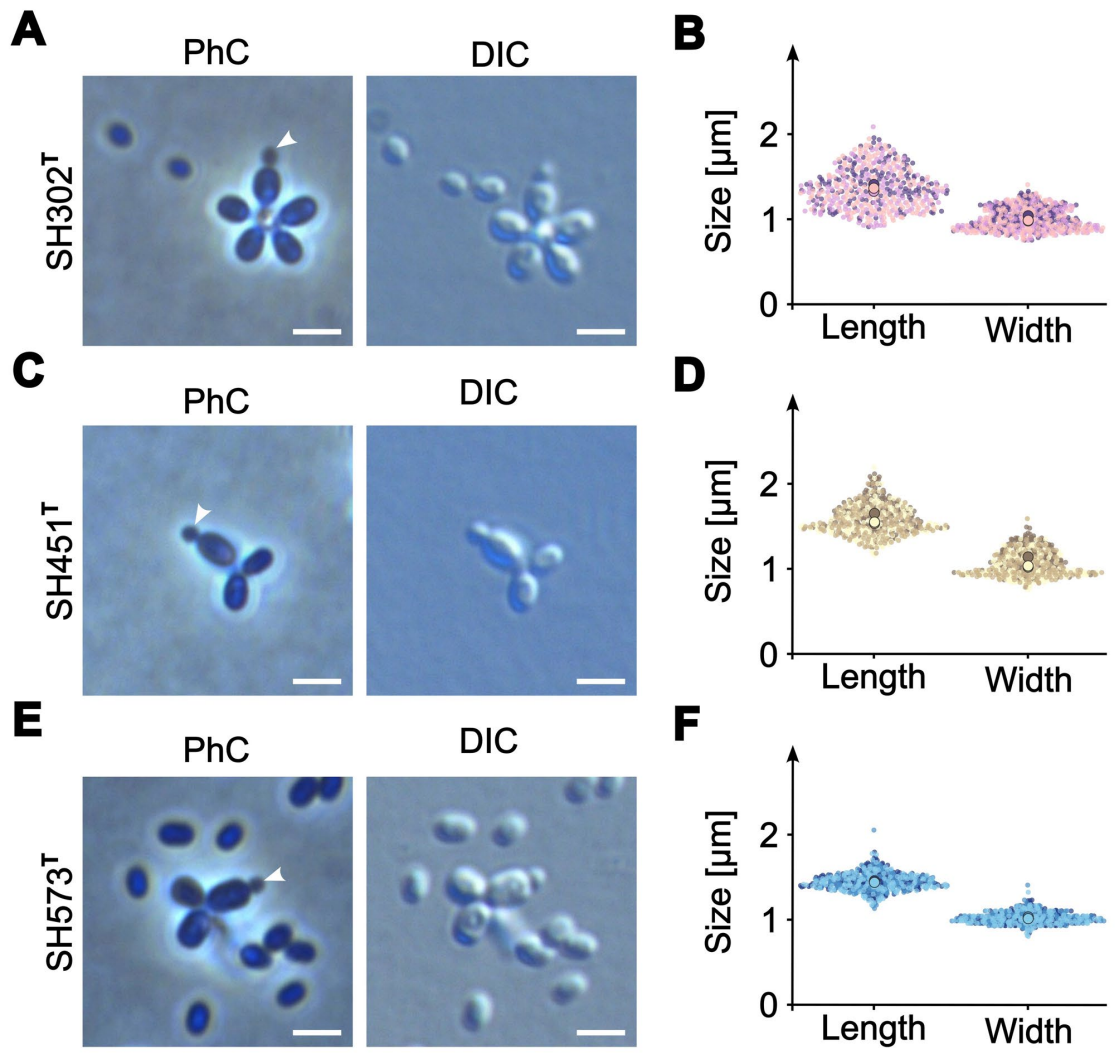
Cell morphology and cell size of the novel strains. Phase contrast (PhC) and differential interference contrast (DIC) micrographs of strains SH302^T^ (**A**), SH451^T^ (**C**), and SH573^T^ (**E**). Cell division events are indicated by white asterisks, marking small daughter cells emerging from larger mother cells. Cell sizes of strains SH302^T^ (**B**), SH451^T^ (**D**), and SH573^T^ (**F**). Data was obtained from three biological replicates with 150 cells measured each. Mean values are shown as larger circles for each replicate. The scale bars represent 2 µm.

A detailed comparison of morphological ans physiological characteristics is provided in Table 2. Colonies of strains SH302^T^ and SH451^T^ appear pink, whereas those of strain SH573^T^ are light pink, all exhibit entire margins and a round morphology (Fig. 4A–C). Individual cells of the three strains are ellipsoid to acorn-shaped with comparable cell dimensions (Fig. 5). Strain SH302^T^ has a mean cell length and cell width of 1.4 ± 0.2 μm and 1.0 ± 0.1 μm, respectively. Strains SH451^T^ and SH573^T^ are slightly larger with mean cell lengths of 1.6 ± 0.2 μm and 1.4 ± 0.1 μm, respectively, and widths of 1.1 ± 0.2 μm and 1.0 ± 0.1 μm, respectively. Cells of all three strains are smaller than those of their closest known relative *A. helgolandensis* Q31a^T^, which measures 1.9 μm in length and 1.0 μm in width^66^. Rosette formation was observed for strains SH302^T^ and SH573^T^, with strain SH573^T^ forming larger rosettes. This trait was also previously reported for strain *A. helgolandensis* Q31a^T^^66^. Consistent with strains belonging to the class *Planctomycetia*, all three strains divide asymmetrically in a process in which a smaller daughter cell emerges on one pole of the larger mother cell (polar budding). The daughter cell grows over time and eventually separates from the mother cell (Fig. 5).

**Table 2:** Phenotypic and physiological features of strains SH302^T^, SH451^T^ and SH573^T^ compared to *Aureliella helgolandensis* Q31a^T^.

Characteristics	SH302^T^	SH451^T^	SH573^T^	*A. helgolandensis* Q31a^T^^66^
Sampling information				
Location	Chalk mine, Lägerdorf, Schleswig–Holstein, Germany	Fjord Schlei, an estuary of the Baltic Sea	Creek Kopendorfer Au (Fehmarn Island, Germany)	Shore at Helgoland Island, Germany
Sampled material	Surface water	Surface water	Surface water	Dead common jellyfish (*Aurelia aurita*)
Phenotypic features				
Pigmentation	Pink	Pink	Light pink	Lucid white
Cell shape	Ellipsoid/acorn-shaped	Ellpsoid/acorn-shaped	Ellipoid/acorn-shaped	Acorn-shaped
Size (length x width) (µm)	1.4 ± 0.2 × 1.0 ± 0.1	1.6 ± 0.2 × 1.1 ± 0.2	1.4 ± 0.1 × 1.0 ± 0.1	1.9 ± 0.2 × 1.0 ± 0.2
Cell division mode	Polar budding	Polar budding	Polar budding	Polar budding
Temperature range (optimum) (°C)	18–32 (28)	21–28 (28)	18–32 (28)	10–33 (27)
pH range (optimum)	7.0–10.0 (7.0)	7.0–10.0 (8.0)	6.0–9.0 (7.5)	6.0–8.0 (7.5)
Relation to oxygen	Aerobic	Aerobic	Aerobic	Aerobic
Stalks	n.o.	n.o.	n.o.	Yes
Aggregates	Yes, rosette formation	n.o.	Yes, rosette formation	Yes

n.o. not observed, n/a data not available.

Growth experiments revealed that strain SH302^T^ grows aerobically at 18–32 °C (optimum 28 °C) and tolerates an external pH of 7.0–10.0 (optimum pH 7.0) (Fig. 4). Strain SH451^T^ exhibits a narrower temperature range of 21–28 °C and grows at pH 7.0–10.0, showing optimal growth at 28 °C and pH 8.0. Strain SH573^T^ also grows between 18–32 °C. However, it tolerates a lower pH range of 6.0 to 9.0 with optimal growth at 28 °C and pH 7.5. These morphological and phenotypic characteristics differ from *A. helgolandensis* Q31a^T^, which exhibits lucid white colonies and grows over a broader temperature range (10–33 °C) and a narrower pH range (6.0–8.0), with optimal growth at 27 °C and pH 7.5^66^.

## Conclusions

Based on the collected data on phenotypic and genomic features, we conclude that the novel strains belong the three novel species. Two species belong to the same genus, the third species belongs to a separate genus. The novel genera are clearly separated from the closest relative *A. helgolandensis* Q31a^T^ as the result of the phenotypic analysis along with phenotypic characteristics, e.g. pigmentation of the colonies. For the novel taxa we introduce the names as mentioned in the protologues provided below.

### Description of *Njordella* gen. nov.

*Njordella* (Njor.del`la. N.L. fem. n. *Njordella*, named after the Norse god Njord, deity of the sea and winds).

Gram-negative cells with aerobic, mesophilic, neutrophilic to slightly alkaliphilic and heterotrophic lifestyle. Cells form pink colonies, are acorn-shaped and reproduce by polar budding. The DNA G + C content is in the range of 50–52%. The genus belongs to the family *Pirellulaceae*, order *Pirellulales*, class *Planctomycetia*, phylum *Planctomycetota*. The type species is *Njordella aestuarii*.

### Description of *Njordella aestuarii* sp. nov.

*Njordella aestuarii* (aes.tu.a’ri.i. L. gen. neut. n. *aestuarii*, of an estuary, referring to the isolation of the type strain from an estuary of the Baltic Sea).

In addition to the features mentioned for the genus, cells have a mean length of 1.6 ± 0.2 µm and a mean width of 1.1 ± 0.2 µm. The type strain is SH451^T^ (= CECT 30907^T^ = KCTC 102089^T^) and was isolated from Fjord Schlei, a 42 km estuary of the Baltic Sea in Northern Germany. The type strain grows optimally at 28 °C and pH 8.0, but growth is observed over a range of 21–28 °C and pH 7.0–10.0. The type strain has a genome size of 6,566,063 bp with a DNA G + C content of 50.9% and lacks extrachromosomal elements.

### Description of *Njordella calcicola* sp. nov.

*Njordella calcicola* (cal.ci’co.la*. *L. masc./fem. n. *calx* (gen. *calcis*), chalk; L. masc./fem. n. suff. *-cola*, an inhabitant; from L. masc./fem. n. *incola*, dweller; N.L. fem. n. *calcicola*, referring to the chalk mine from which the type strain was isolated).

In addition to the features mentioned for the genus, cells measure 1.4 ± 0.2 µm length and 1.0 ± 0.1 µm width. Cells are ellipsoid to acorn-shaped and form rosettes. The type strain is SH302^T^ (= DSM 116728^T^ = KCTC 102091^T^) and was isolated from water in a chalk mine in Lägerdorf in the surroundings of Hamburg in Northern Germany. Cells of the type strain grow under aerobic conditions at 18–32 °C (optimum 28 °C) and tolerate external pH values of 7.0–10.0 (optimum pH 7.0). The genome of the type strain has a size of 7,174,608 bp and a DNA G + C content of 52.1%. Extrachromosomal elements may be present.

### Description of *Rania* gen. nov.

*Rania* (Ra’ni.a. N.L. fem. N. *Rania*, named after the Norse Sea goddess Rán who rules over the ocean and its treasures).

Cells are Gram-negative, aerobic, mesophilic, neutrophilic and heterotrophic. Colonies are pink-pigmented and acorn-shaped, reproducing by polar budding. Cells typically aggregate in rosettes. The genus belongs to the family *Pirellulaceae*, order *Pirellulales*, class *Planctomycetia*, phylum *Planctomycetota*. The DNA G + C content is around 54–55%. The type species is *Rania fehmarnensis*.

### Description of *Rania fehmarnensis* sp. nov.

*Rania fehmarnensis* (feh.mar.nen’sis. L. fem. adj. *fehmarnensis*, referring to Fehmarn Island in the Baltic Sea, from which the type strain was isolated).

Cells measure 1.4 ± 0.1 µm in length and 1.0 ± 0.1 µm in width. Cells forms rosettes. The type strain is SH573^T^ (= DSM 116763^T^ = KCTC 102135^T^ = CECT 31080^T^) and was isolated from the creek Kopendorfer Au on Fehmarn Island in the Baltic Sea. The type strain shows growth at temperatures between 18–32 °C (optimum 28 °C) and at pH 6.0–9.0 (optimum pH 7.5) under aerobic conditions. The type strain genome has a size of 9,357,046 bp, a DNA G + C content of 54.6% and lacks extrachromosomal elements.

## Supplementary Information

Below is the link to the electronic supplementary material.

Supplementary Material 1

## Data Availability

The 16S rRNA gene sequences of the novel isolates are available from GenBank under the accession numbers PX530357 (SH302^T^), PX530377 (SH451^T^) and PX530413 (SH573^T^). Genome sequences have been deposited at DDBJ/ENA/GenBank under the accession numbers JBWOZO000000000 (SH302^T^), JBWOZP000000000 (SH451^T^) and JBWOZQ000000000 (SH573^T^).
